# Artificial Intelligence-Based Computational Screening and Functional Assays Identify Candidate Small Molecule Antagonists of PTPmu-Dependent Adhesion

**DOI:** 10.3390/ijms24054274

**Published:** 2023-02-21

**Authors:** Kathleen Molyneaux, Christian Laggner, Susann M. Brady-Kalnay

**Affiliations:** 1 Department of Molecular Biology & Microbiology, School of Medicine, Case Western Reserve University, 10900 Euclid Ave., Cleveland, OH 44106-4960, USA; 2 Atomwise Inc., 717 Market St., San Francisco, CA 94103, USA

**Keywords:** cancer, glioblastoma, PTPmu, receptor protein tyrosine phosphatase, cell adhesion, drug discovery, artificial intelligence drug design

## Abstract

PTPmu (PTPµ) is a member of the receptor protein tyrosine phosphatase IIb family that participates in cell-cell adhesion and signaling. PTPmu is proteolytically downregulated in glioblastoma (glioma), and the resulting extracellular and intracellular fragments are believed to stimulate cancer cell growth and/or migration. Therefore, drugs targeting these fragments may have therapeutic potential. Here, we used the AtomNet^®^ platform, the first deep learning neural network for drug design and discovery, to screen a molecular library of several million compounds and identified 76 candidates predicted to interact with a groove between the MAM and Ig extracellular domains required for PTPmu-mediated cell adhesion. These candidates were screened in two cell-based assays: PTPmu-dependent aggregation of Sf9 cells and a tumor growth assay where glioma cells grow in three-dimensional spheres. Four compounds inhibited PTPmu-mediated aggregation of Sf9 cells, six compounds inhibited glioma sphere formation/growth, while two priority compounds were effective in both assays. The stronger of these two compounds inhibited PTPmu aggregation in Sf9 cells and inhibited glioma sphere formation down to 25 micromolar. Additionally, this compound was able to inhibit the aggregation of beads coated with an extracellular fragment of PTPmu, directly demonstrating an interaction. This compound presents an interesting starting point for the development of PTPmu-targeting agents for treating cancer including glioblastoma.

## 1. Introduction

Glioblastoma is the most common, aggressive, and lethal type of glioma, which is a primary brain tumor that arises from stem cells of the brain [1,2]. Considering the poor postoperative survival time of glioblastoma (12–14 months) [1,2], new therapies are urgently needed and can be achieved by targeting proteins, such as the receptor tyrosine phosphatase mu (PTPµ), known to regulate the behavior of these tumor cells.

PTPµ is a prototypical member of the RPTP IIb family [3,4]. The extracellular domain of PTPµ mediates homophilic cell-cell adhesion [5,6,7], and its intracellular domain mediates signaling via protein tyrosine phosphatase activity [8,9,10,11,12,13]. PTPµ is proteolytically downregulated in glioblastoma [14], and high-grade gliomas have very little full-length PTPµ protein [15]. However, both extracellular [16] and intracellular [14] fragments of PTPµ accumulate in the tumor.

The PTPµ fragments present in gliomas are hypothesized to have oncogenic potential as it has been shown that knocking down PTPµ in tumor cells decreases their invasive and migratory behaviors as wells as their ability to form tumors in rodent xenograft models of human glioma [15,17]. Conceivably, targeting either the adhesive or enzymatic function of PTPµ could generate therapeutic agents, but the phosphatase catalytic domain is highly charged, meaning strong interacting agents are unlikely to be cell permeable [18]. There are also many tyrosine phosphatases (both receptor and non-receptor) that have structurally similar enzymatic domains, meaning agents targeted to the active site could be promiscuous.

Receptor tyrosine phosphatase family members do, however, have divergent extracellular domains [4,7,19,20], creating a unique opportunity for drug design [21]. The extracellular domain of PTPµ exhibits only 49–63% amino acid identity with other RPTPIIb family members [22], and its functional domains are well characterized [5,6,23]. They consist of a MAM domain, unique to the IIb RPTP subtypes, an Ig domain, and four fibronectin three (FNIII) repeats. The Ig domain has been shown to be necessary and sufficient to mediate the binding of beads to PTPµ-coated surfaces [6], while the MAM domain may participate in both cis and trans interactions [24,25,26,27]. Domain swapping experiments and identification of tumor-derived mutations in RPTPIIb proteins have further characterized the adhesive function of this subfamily [7,28,29]. In addition, a systematic analysis of deletion constructs in an Sf9-expression system revealed that the MAM, Ig, and first two FNIII repeats comprise the smallest functional unit for efficient cell-cell adhesive activity [22]. A crystal structure of a fragment comprising the MAM, Ig, and FNIII-1, and FNIII-2 domains indicates that two protein chains form a homophilic trans (antiparallel) dimer, with the MAM and Ig domains of one molecule interacting with the FNIII-1 and FNIII-2 domains of another molecule [26].

This crystal structure informed the development of peptides designed to interact with surface exposed loops in the MAM and Ig regions [16]. In mouse orthotopic tumor models, these agents have been shown to bind to extracellular fragments of PTPµ that accumulate in the tumor microenvironment and detect tumors including both tumor margins and cells migrating away from the main tumor mass [16,30]. These peptides are being developed as imaging agents and could also be exploited for drug delivery [16,30,31,32,33,34,35,36]. However, small molecule targeting agents are orally deliverable and typically metabolically stable making them easier to use than peptide-based drugs. Thus, we utilized AtomNet^®^ [37], the first deep learning neural network for structure-based drug design and discovery, developed by Atomwise Inc. to identify small molecules predicted to interact with the extracellular domain of PTPµ and tested them in two cell-based assays: PTPµ-mediated Sf9 aggregation as well as glioma cell sphere formation and three-dimensional (3D) survival and growth. Growth in 3D culture is a characteristic of tumor cells and can be used as a surrogate measure for tumor formation [38]. Our top hit was confirmed to affect PTPµ activity in an in vitro bead-based adhesion assay, indicating the effectiveness of this modeling and screening approach.

## 2. Results

### 2.1. Atomwise Virtual Screen

**Figure 1:**
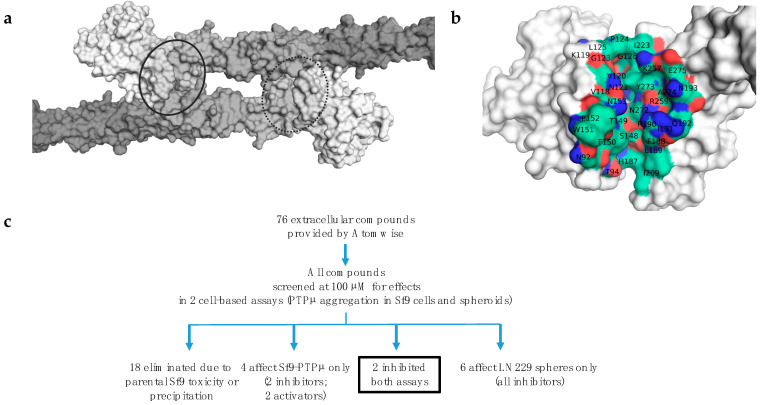
Computational and functional screening strategy. (**a**) Shape overview of the PTPµ trans homodimer (PDB ID 2V5Y, white to gray with increasing residue number), with the location of the targeted binding sites circled. (**b**) Closeup of a monomer chain (region circled in (**a**)), showing the cleft between the MAM (left side, residues 22–184) and Ig (right side, residues 186–277) domains that was targeted as a potential ligand binding site. Binding site residues are shown in green, red, and blue for carbon, oxygen, and nitrogen atoms, respectively, with labels for the relevant amino acid residues. The residues were renumbered from the original PDB file to match the canonical sequence for UniProt ID P28827. (**c**) Functional screening approach. Atomwise provided 76 potential compounds and two blinded DMSO control samples. These were screened in cell-based assays for PTPµ-mediated aggregation of Sf9 cells and glioma cell (LN229) sphere formation and growth in 3D culture. A third unblinded DMSO sample was used for normalization purposes. Compounds were identified that affected either or both assays.

We investigated the available crystal structures for PTPµ and related proteins to identify suitable binding sites for small molecules. The groove involved in the trans-dimer interaction seen in the crystal structure [26] [Protein Data Bank IDs 2V5Y] is predicted to be the most druggable site on the extracellular domain (ECD) (Figure 1a,b) based on the ICM pocket finder module (v3.8-7, Molsoft L.L.C. (San Diego, CA, USA)), which assigns a druggability score based on the volume of the putative binding site and its hydrophobicity and the degree to which the site is buried [39]. This pocket sits between the MAM and Ig domains and partially overlaps with the homodimer contact site. Therefore, targeting it could restrict flexibility between those two critical domains and prevent certain types of homophilic interactions. Thus, Atomwise selected this region as the target site for virtual high throughput screening.

A single global AtomNet^®^ model, which is a tool for structure-based drug design and predicting protein-ligand interactions [37,40], was used to predict the binding affinity of small molecules to the putative PTPµ drug-binding pocket. The binding constants (i.e., K_i_, K_d_, and IC_50_ values), based on experimental data, the structures of thousands of proteins from various families, and millions of small molecules all derived from curated public databases and proprietary sources, were used to train the model that was then used to screen for novel binding sites and ligands. Because AtomNet^®^ is a single global model, the likelihood of overfitting is reduced. The model training used a three-step procedure: (1) a flooding algorithm based on an initial seed was used to define the binding site [41], with the seed derived from either bound ligands annotated in the PDB database, previously reported catalytic motifs, or critical residues revealed through mutagenesis; (2) the coordinates of the protein-ligand complex were translated into a 3D Cartesian space with the origin defined as the center-of-mass of the binding site. The protein structure was randomly rotated and translated around the center-of-mass of the binding site to prevent the neural network from memorizing a preferred structural orientation; (3) for each ligand, multiple poses, each representing a putative protein-ligand complex, within the binding site were sampled, which means that our method does not require experimental co-complexes for either training or prediction. Uniformly-sized regular 3D grids were generated by rasterizing each co-complex, with grid point values representing the presence of different atom types at that point, similar to how photo imaging assigns pixels into red, green, and blue channels. The receptive field of our convolutional neural network is defined by these grids. The network architecture was as described in Hsieh et al. [42]. Final scores for each binding pose were calculated through weighted Boltzmann averaging and compared to the experimentally measured pK_d_, pK_i,_ or pIC_50_ of the protein and ligand pair. A root-mean-square-error loss function was used to adjust the weights of the neural network, thus, reducing the error between predicted and experimentally measured affinities. Training was done with the ADAM [43] adaptive learning method using the backpropagation algorithm and mini-batches with 64 examples per gradient step.

The trained AtomNet^®^ model was used to score and rank compounds from the 20180722 version of the Mcule library of commercially available small drug-like molecules (6.8 M compounds after pre-processing, https://mcule.com/, accessed on 1 October 2018) using an ensemble of ligand-protein conformations. The preparation of the screening library as well as the post-processing of the top-scoring molecules has been described in detail before [42]. The 30,000 top-scoring compounds were filtered to remove compounds that were predicted to be insoluble by Atomwise’s solubility model or contained undesired (potentially reactive, unstable, or promiscuous) chemical moieties. The Butina clustering algorithm [44], which selects compounds by going down a ranked list and keeping only those entries that meet a certain diversity cutoff (here: ECFP4 fingerprint similarity with a Tanimoto coefficient ≥ 0.4) was employed to arrive at a final subset of 77 deliverable compounds. These compounds were provided together with two blinded DMSO controls.

### 2.2. Functional Screening Strategy

The 76 compounds predicted by computational modeling to interact with the extracellular domain of PTPµ were screened in two cell-based assays: an Sf9 aggregation assay [5] and a glioma cell 3D sphere formation and growth assay [45] (Figure 1c). Two DMSO samples were provided by Atomwise as blinded controls, while a third non-blinded DMSO sample was used as a control for normalization purposes. The Sf9 assay was chosen because it directly tests the adhesive action of PTPµ [5] since Sf9 cells lack endogenous PTPµ (and other RPTPIIb family members: http://ptp.cshl.edu/protein.shtml, accessed on 3 March 2022) and do not normally self-aggregate. However, baculoviral-mediated overexpression of PTPµ drives homophilic adhesion of Sf9 cells [5]. Historically, aggregation assays were performed in glass scintillation vials, which have ideal surface characteristics but are unwieldy for screening purposes. This assay was scaled to a low volume multi-well format to accommodate moderate throughput. We achieved this by adjusting rotation speed and utilizing an affordable and adaptable non-adhesive coating (highly hydrolyzed polyvinyl alcohol) that we developed [45]. Without this coating, cells stick to the plastic (whether tissue-culture treated or not) and fail to aggregate.

We used the same versatile non-adhesive coating to perform a glioma sphere-based assay. This assay was selected to run in parallel because the ultimate goal is to identify compounds that have therapeutic potential against cancers such as glioblastoma. Glioma cells (LN229s) cultured on our non-adhesive coating cluster together and grow as 3D structures that can model some of the complexity of the tumor microenvironment. In particular, a peptide imaging agent that recognizes the extracellular domain of PTPµ labels the central core of these spheres [45], indicating the possible accumulation of cleaved fragments, similar to what is observed in actual tumors.

Eighteen compounds were eliminated due to either general toxicity or poor solubility. One compound was found to be toxic to parental Sf9 cells, indicating an off-target effect as these cells lack expression of PTPµ (Supplemental Figure S1), and 17 compounds were eliminated due to insolubility under the conditions used for the Sf9 aggregation assay and/or the 3D sphere assay (Supplemental Figure S2). The precipitates appeared as fine dust accumulating around the spheres (due to the u-bottom shape of the plate), or as specks, needles, scattered debris, etc.

Of the remaining 58 compounds, four affected PTPµ aggregation only, six affected glioma 3D spheres (aggregation and/or growth) only, and two, our priority compounds, were inhibitors in both assays. The predicted Gibb’s free energy for association of 0205629321 was −6.0. The predicted Gibb’s free energy for 0205603181 was −6.3. Carbon-substituted base structures and binding poses of the two priority compounds are shown in Supplemental Figures S3 and S4.

### 2.3. PTPµ-Dependent Sf9 Aggregation

**Figure 2:**
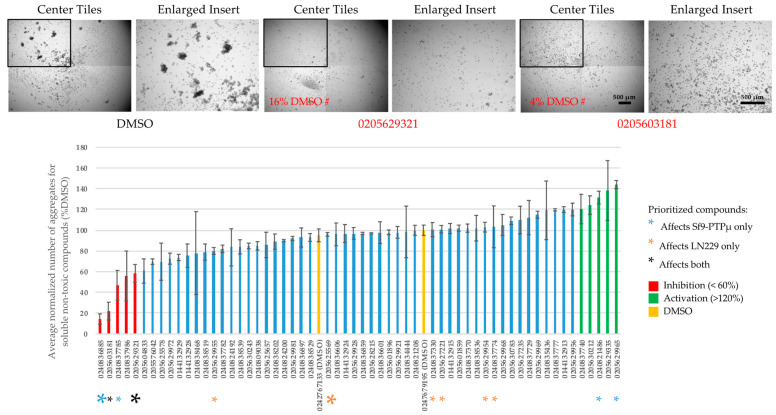
Functional screening identifies compounds able to affect PTPµ-mediated aggregation of Sf9 cells. PTPµ-expressing Sf9 cells were treated with the indicated compounds (100 µM) for 20 min then rotated for 30 min to stimulate aggregation. (**Top**): images of samples treated with priority compounds. Each well was imaged in its entirety as a 4 × 4 grid, but just central tiles are shown. The examples show the replicate with the strongest effect (0205629321: n = 8 and 0205603181: n = 4). (**Bottom**): the number of aggregates with footprint areas over 4000 µm^2^ were counted and normalized to the average number present in the un-blinded DMSO control. The compound barcodes are shown on the x-axis. Inhibitors (<60%), activators (>120%), and the positions of the blinded DMSO samples are indicated. Colored asterisks indicate the functional categories of the compounds. Large asterisks indicate the top hit in each category. Values are averages ± standard errors of the means (s.e.m). Samples that showed no effect in the initial screen (n = 2). Priority compounds n = 4–8 replicates.

Figure 2 shows examples of the effects of our priority compounds (bar codes 0205629321, 0205603181) on PTPµ-mediated aggregation of Sf9 cells. Aggregates are readily apparent in control samples, but wells treated with putative PTPµ inhibitors contain mostly single cells or small clusters.

The effects of the compounds on PTPµ-dependent aggregation were quantified by counting the total number of aggregates above 4000 µm^2^ in each well and normalizing that to the average number present in the un-blinded vehicle control wells. Figure 2 shows the results for all the soluble non-toxic compounds. In addition to our two priority compounds, we identified compounds that affected PTPµ aggregation only (positions indicated by color-coded asterisks). The strongest two inhibitors (0240836885 and 0240837785) and one activator (0240821486) in this category were selected for further analysis. Compounds exhibiting poor reproducibility between replicates were rejected. As indicated in Figure 1, we also identified compounds that affected tumor spheres without affecting PTPµ aggregation, and the positions of these compounds (color-coded asterisks) are indicated for reference.

**Figure 3:**
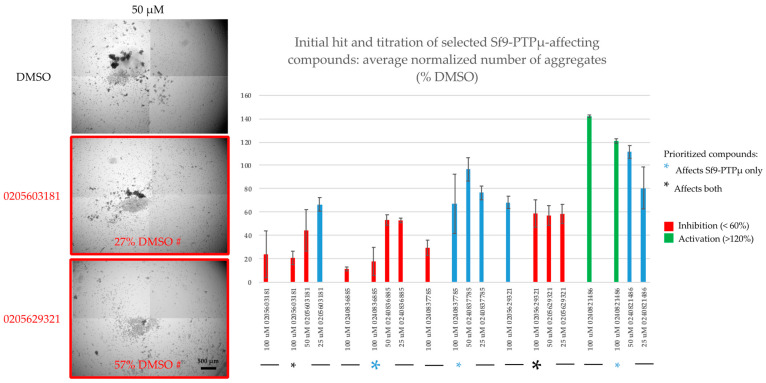
Titration of selected compounds in the PTPµ aggregation assay in Sf9 cells. PTPµ-expressing cells were treated with the indicated doses of compounds then rotated to stimulate aggregation. (**Left**): examples of the effects of our two priority compounds at an intermediate dose (50 µM). Each well was imaged in its entirety as a 4 × 4 grid, but just representative central tiles are shown. (**Right**): quantitation of the dose-dependent effects of selected compounds on PTPµ aggregation in Sf9 cells. PTPµ-expressing cells were treated with the indicated doses of compounds then rotated to stimulate aggregation. The number of aggregates were counted and normalized to the average number present in the matched DMSO control. The initial hit (at 100 µM; n = 2 replicates) and independent follow-up titration results (25–100 µM) are shown. Colored asterisks indicate the functional categories of the compounds. The large asterisks indicate the top hit in each category. Values are averages (n = 2 replicates) ± s.e.m.

Our initial screen was performed at 100 µM, but to identify the most potent agents we titrated our two priority compounds and other selected hits (Figure 3 and Supplemental Figures S5 and S6). Of the two priority inhibitors, only one (0205629321) was active down to 25 µM and in fact, exhibited the same degree of inhibition (~60%) at all tested doses. This could indicate that we have saturated binding with this compound at all tested concentrations, and that even with 100% occupancy the compound cannot fully abolish the adhesive activity of PTPµ. It is likely that its activity will taper at lower doses, but these have not yet been tested. Of the Sf9-PTPµ-only inhibitors, one compound (0240836885, the strongest identified in Figure 2) was also active down to 25 µM (Figure 3). The one tested activator was weaker at 100 µM on follow-up and was not effective at lower doses (Figure 3 and Supplemental Figure S6).

### 2.4. Glioma Sphere Formation and Growth

**Figure 4:**
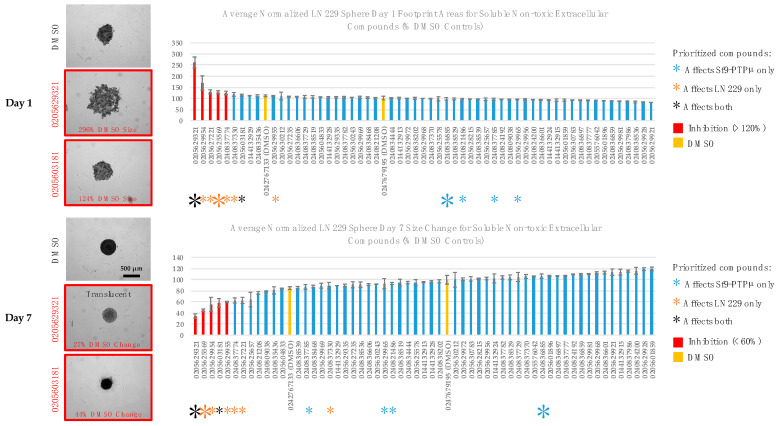
Functional screening identifies compounds able to inhibit glioma sphere formation and growth. LN229 cells were plated into non-adherent 96-well u-bottom plates and treated with compounds (100 µM) for 7 days. (**Left**): representative images of day 1 and day 7 spheres treated with the priority compounds (100 µM). On day 1, a larger footprint size indicates that sphere formation was slowed. On day 7, a smaller size change indicates impaired growth. (**Right**): quantitation of the effects of all soluble non-toxic compounds (100 µM) on sphere formation and growth. Sphere footprint sizes were measured on day 1 and normalized to the average footprint size of the un-blinded DMSO controls. Sphere footprint sizes were measured again on day 7 and the % size change calculated and normalized to the average size change of the controls. Inhibitors and the positions of the blinded DMSO samples are indicated. Colored asterisks indicate the functional categories of the compounds. The large asterisks indicate the top hit in each category. Values are averages ± s.e.m. Samples that showed no effect in the initial screen (n = 2). Priority compounds n = 4–8 replicates.

When glioma cells are cultured on non-adherent surfaces, they compact into an aggregate and grow as a 3D structure [45]. We tested all soluble non-toxic compounds in this assay, and Figure 4 shows representative effects of our two priority compounds on glioma cell aggregation and consolidation (day 1) and sphere growth (day 7). The two compounds slowed both of these processes, resulting in a broader spread of cells on day 1 and smaller spheres by day 7. One of the priority compounds (0205629321) also produced an interesting qualitative change in the appearance of the day 7 spheres. These spheres were translucent instead of optically dense.

To quantify sphere aggregation, we measured the day 1 footprint areas of the spheres and normalized that to the area of the vehicle-treated controls (Figure 4). To quantify sphere growth, we determined the change in footprint size between day 1 and day 7 and normalized that to the size change of the controls (Figure 4). Of our two priority compounds (color-coded asterisks), the one with the most potent effect on PTPµ aggregation (0205629321) based on it retaining activity down to 25 µM (Figure 3) was also the strongest in these assays. The other priority inhibitor (0205603181) affected sphere growth but not aggregation. As indicated in Figure 1, there were also six compounds that were ineffective in the PTPµ aggregation assay but behaved as inhibitors of glioma sphere condensation and/or growth (color-coded asterisks in Figure 4). The positions of the four compounds that affected PTPµ aggregation but not glioma spheres are also shown for reference.

**Figure 5:**
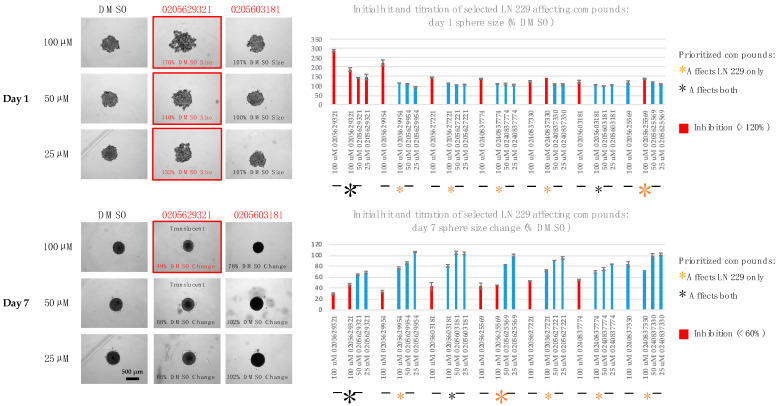
Titration of the effects of selected compounds on sphere formation and growth. LN229 cells were plated into non-adherent 96-well u-bottom plates and treated with the indicated doses of compounds for 7 days. (**Left**): representative images of day 1 and day 7 spheres treated with the two priority compounds. (**Right**): quantitation of the dose-dependent effects of selected compounds on glioma sphere formation and growth. Sphere footprint sizes were measured on day 1 and normalized to the average footprint size of the un-blinded DMSO controls. Sphere footprint sizes were measured again on day 7 and the % size change calculated and normalized to the average size change of the controls. The initial hit (at 100 µM) and independent follow-up titration results (25–100 µM) are shown. Colored asterisks indicate the functional category of the compound. The large asterisks indicate the top hit in each category. Values are averages (n = 2 replicates) ± s.e.m.

We titrated the priority compounds (Figure 5) and five select glioma sphere-only inhibitors (Figure 5, and Supplemental Figure S7) to identify the compounds with the most potent effects on glioma cells. Our best priority candidate (0205629321) affected sphere aggregation down to 25 µM but was only effective on sphere growth at 100 µM. A change in sphere opacity was, however, observed down to 25 µM. The other priority compound did not affect sphere condensation in follow-up and had only very modest effects on sphere growth. The compounds identified as affecting glioma spheres only were either weak or lacked activity in follow-up. Two of these compounds did, however, cause interesting qualitative changes in the day 7 appearance of spheres (Supplemental Figure S7). Spheres exposed to 25 µM of one of these compounds (0205625569) were ovoid instead of round. In addition, one compound (0240837330) appeared to cause sloughing of cells from the surface of spheres when used at both 50 µM and 100 µM. Based on Helix Blue staining, this compound was not acutely toxic (at 24 h of treatment) (Supplemental Figure S1) but the sloughing of cells on day 7 could indicate long-term toxicity.

### 2.5. Bead Assay to Assess Direct Interactions between Selected Compounds and PTPµ

**Figure 6:**
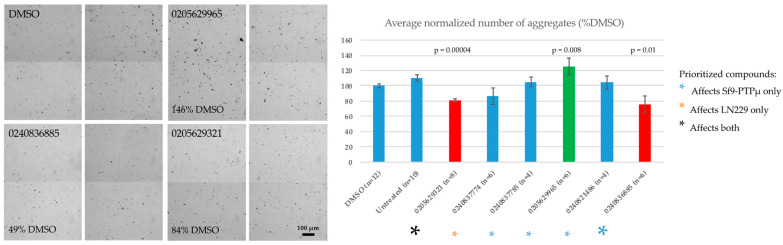
The effect of selected compounds in a bead-based aggregation assay. MonoMag streptavidin beads coated with PTPµ21-365avi were treated with 100 µM compounds for 20 min and then induced to aggregate by rotation. The number of aggregates over 50 µm^2^ in 16 frames per well were counted and normalized to the average number present in the vehicle-treated control wells. Colored asterisks indicate the functional category of the compound. Values are averages ± s.e.m. Significance was tested with Student’s *t*-test. *p*-values are shown for those compounds found to cause a statistically significant effect (*p* < 0.05) on aggregation (relative to the DMSO control).

Because off-target effects are possible in cell-based assays, we tested our priority compounds (that affected both PTPµ aggregation in Sf9 cells and inhibited glioma spheroids) and select compounds that just affected PTPµ aggregation in Sf9 cells in a PTPµ-mediated bead aggregation assay (Figure 6). One weak compound from the glioma-only category was tested for comparison. Streptavidin beads were coated with a bacterially-expressed avi-tagged fragment of PTPµ containing the MAM, Ig, and first FNIII repeat (PTPµ21-365avi). Coated beads were treated with compounds (100 µM) for 20 min and then induced to aggregate by rotation. One of our priority inhibitors (0205629321) had a modest, but statistically significant, effect on bead aggregation. The other priority inhibitor was not soluble in the buffer used for this assay (PBS + 0.01% Tween) despite being soluble in media. Two compounds from the Sf9-only category also had statistically significant effects: 0240836885 inhibited and 0205629965 activated bead aggregation. Notably, 0240836885 was the strongest inhibitor and 0205629965 the strongest activator of Sf9-PTPµ-aggregation identified in the screen (Figure 2).

## 3. Discussion

Using AI-targeted drug design and cell-based functional screens, we identified compounds able to perturb PTPµ-dependent adhesion and/or affect glioma cell growth in 3D culture. The PTPµ aggregation assay is a direct test of PTPµ-mediated aggregation, as parental Sf9 cells lack PTPµ, so compounds affecting this assay are more likely to be specific targeting agents; however, compounds affecting spheres are more likely to have therapeutic potential. The fact that we obtained inhibitory compounds in the sphere assay that produced qualitatively different effects (i.e., changes in optical appearance and shape or sloughing of cells) indicates that different processes are being perturbed. The “clearing” effect of the priority compound that acted as an inhibitor of both PTPµ-dependent Sf9 aggregation and bead aggregation may serve as a useful benchmark for what to expect when PTPµ-dependent adhesion is perturbed in 3D cancer spheroids. This can streamline testing additional candidates, particularly those identified by structure activity relationship analysis based upon these original hits.

Drug design has focused largely on biochemical assays to identify agents able to affect enzymes, most often kinases [46]. This is due to the ease of assay design. However, considering the important role of cell-cell and cell-substrate adhesion in development and disease processes there is considerable interest in drug development in this space. At the moment, the majority of such agents being tested are antibody or peptide-based [47,48]. Peptides, in particular, have stimulated the design of small molecule mimetics [49,50], including pioneering work to identify antagonists and agonists of N-cadherin mediated adhesion [51,52], which may be of clinical utility in inhibiting cancer growth and supporting nerve regrowth after injury, respectively. Thus, studies to identify peptide mimetics may lead to the development of more convenient orally available drugs for cancer and other conditions.

Identifying agents able to affect the complicated interactions required for efficient adhesion (which in the case of PTPµ involves multiple domains and both trans [5,6,7,22,26,28,29] and cis [25] interactions) is not straightforward. Biochemical assays using fragments cannot model the kinds of interactions seen with the full-length protein in the context of a cell membrane. In this sense, it is surprising, and impressive, that we were able to identify an agent effective in both cell and bead-based binding assays, considering we were targeting a single binding pocket in what is likely a large adhesive interface. However, the agents that affected PTPµ aggregation in Sf9 cells, but not bead aggregation, are still worth pursuing as their inhibition of PTPµ may be more conformationally sensitive. Their structures can inform how to improve the interaction between a compound and PTPµ, and structure-activity relationship studies might yield compounds with higher affinity or better activity in cancer-cell assays. Additionally, small molecules able to interact with PTPµ could be further derivatized to serve as both imaging and therapeutic agents (i.e., theranostics). These could be useful agents for treating glioblastoma and other cancers (breast, prostate, lung, ovarian, endometrial, and melanoma [53]) where PTPµ is proteolytically dysregulated.

In conclusion, we have identified small PTPµ-targeting molecules that have therapeutic potential. Future work will focus on whether these compounds affect PTPµ-dependent cancer cell behaviors (e.g., cell migration, invasion, or proliferation) in vitro and in vivo. Finally, it would be interesting to test whether these compounds alter the catalytic activity of PTPµ considering changes in adhesion are likely translated into changes in signaling. In this manner, we may have been able to take advantage of the outside-in approach [21], bypassing some of the challenges associated with directly targeting the enzymatic active site of PTPµ. It is worth noting that drug design for tyrosine phosphatases has mostly pivoted to trying to identify allosteric inhibitors by targeting regulatory regions away from the catalytic site [54,55]. For PTPµ (and other RTP IIb family members) this could involve targeting a juxtamembrane regulatory motif called the wedge domain that is thought to regulate enzymatic activity by controlling complex inter- or intra- molecular interactions [56]. A peptide agent targeting the wedge domain of PTPµ has been shown to block glioma cell migration [14], and its structure could inform future screens.

## 4. Materials and Methods

### 4.1. PTPµ-Dependent Sf9 Aggregation Assays

This assay tests cell-cell adhesion or aggregation of cells induced by PTPµ expression. 48-well culture plates (Corning #353078, Corning, NY, USA) were treated with 0.75% (wt/vol) PVA (Sigma-Aldrich, >99+% hydrolyzed, number-average MW ca. 130 kD) to create a unique non-adhesive substrate [45]. Sf9 cells from ATCC cultured in Grace’s Complete Medium (Gibco, Grand Island, NY, USA) + 10% FBS (HyClone, South Logan, UT, USA) were infected with baculovirus to express human full-length PTPµ [8]. Approximately 40 h after infection, cells were resuspended in the culture media (which includes floating cell clumps detached during infection) and gently triturated to create a single cell suspension. Cells were counted and diluted to a concentration of 12.8 × 10^4^ cells/mL, and 90 µL (1.15 × 10^4^) were plated per non-adherent well. To give the indicated final concentrations, 90 µL of compound (prepared in media) was added to the wells (2× replicates per compound). Bubbles (which interfere with aggregation) were removed by puffing air across the plate with an empty squeeze bottle. Plates were incubated at room temperature for 20 min, and aggregation was induced by rotation at 120 rpm on an orbital shaker. Plates were manually shaken to distribute aggregates away from the center of the wells; then, each well was imaged in its entirety by capturing a 4 × 4 grid at 5× magnification on a Leica CTR6500 microscope fitted with an automated stage.

### 4.2. Glioma Sphere Assays

The same unique PVA substrate described above was used to induce formation of 3D tumor spheroids [45]. As previously described, 96-well u-bottom non-tissue culture-treated plates were coated with 0.75% (wt/vol) PVA and allowed to dry upside down. LN229 cells, obtained from ATCC, were cultured to confluence in DMEM (High Glucose DMEM (Gibco, Grand Island, NY, USA) + 5% FBS (HyClone, South Logan, UT, USA), trypsinized, and resuspended at a plating concentration of 15 × 10^4^ cells/mL, and 50 µL (7500 cells) plated per well into the internal wells of the prepared plate. To give the indicated final concentrations, 50 µL of compound (prepared in media) was added to the wells (2× replicates per compound) and 100 µL of PBS was plated per well into the external wells of the plate (to buffer edge effects), and the plate was cultured for 7 days at 37 °C and 5% CO_2_. Spheres were imaged at 5× magnification using a Leica CTR6500 microscope fitted with an automated stage.

### 4.3. Bead Aggregation Assay

Bead aggregation assays were performed in 48-well plates pretreated with anti-adherence rinse (Stemcell Technologies, Vancouver, CA, USA) per the manufacturer’s instructions. A bacterially expressed biotin-tagged fragment of PTPµ corresponding to aa 21-365 (PTPµ21_365avi—containing the MAM, Ig, and first FNIII repeat) was diluted into PBS + 0.01% Tween 20 to 30 ng/µL. Streptavidin MonoMag beads (1 micron diameter, Ocean NanoTech, San Diego, CA, USA) were added at a ratio of 53 ng protein/µg of beads and incubated at room temperature for 15 min. Coated beads were diluted to 6 µg beads/mL (~1.6 × 10^7^ particles/mL) in PBS + 0.01% Tween 20, and 90 µL used per well. Compounds were diluted into PBS + 0.01% Tween 20 and 90 µL added per well (2 × replicates) to give a final concentration of 100 µM. The plate was incubated at room temperature for 20 min; then, aggregation was induced by rotation at 150 rpm for 30 min. The plate was manually shaken to randomly distribute aggregates, and sixteen 20× images (a 4 × 4 grid with 1000 micron spacing in both x- and y-axes) captured per well on a Leica CTR6500 microscope fitted with an automated stage. This does not tile the entire well, but captures a sampling to account for the random distribution of particles.

### 4.4. Image Analysis

The number of Sf9 aggregates over an arbitrary footprint-size threshold of 4000 µm^2^ were quantified using ImageJ (v1.52a http://imagej.nih.gov/ij, accessed on 2 February 2018):

run(“Set Scale...”, “distance = 0.274 known = 1 pixel = 1 unit = microns global”);

run(“Subtract Background...”, “rolling = 1 light”);

run(“Invert”);

run(“Smooth”);

run(“Smooth”);

setAutoThreshold(“Default dark”);

setOption(“BlackBackground”, true);

run(“Convert to Mask”);

run(“Fill Holes”);

run(“Make Binary”);

run(“Analyze Particles...”, “size = 4000–60,000 show = Masks summarize”);

The number of smoothing steps can be adjusted as necessary to yield uniform aggregate outlines. The upper limit of size was applied to eliminate flagging large clusters of loose cells (which often swirl to the center) as an aggregate. This macro was created to accommodate illumination differences between the edge and center of the plate; however manual correction was necessary to detect shadowed aggregates, adjust for touching aggregates or pooling of loose cells, eliminate debris etc.

The footprint size of spheres was measured with ImageJ:

run(“Set Scale...”, “distance = 0.274 known = 1 pixel = 1 unit = microns global”);

setOption(“BlackBackground”, true);

run(“Convert to Mask”);

run(“Analyze Particles...”, “size = 7000-Infinity show = Masks include summarize”);

The number of bead aggregates over an arbitrary footprint size of 50 µm^2^ were quantified with ImageJ:

run(“Set Scale...”, “distance = 1.1 known = 1 unit = micron”);

run(“Subtract Background...”, “rolling = 20 light”);

setOption(“BlackBackground”, true);

run(“Make Binary”);

run(“Analyze Particles...”, “size = 50-Infinity show = Masks summarize”);

Data for each replicate were normalized to the average value of the DMSO controls and then averaged and presented as a percentage + standard error of the mean. The majority of compounds were only assessed with an n of two, but for compounds flagged as active in the initial screen, the n varies from 4–8 replicates at 100 µM and 2 replicates at 50 µM and 25 µM.

## Data Availability

All data and materials are available upon request to S.M.B.-K. (susann.brady-kalnay@case.edu).

## Supplementary Materials

The following supporting information can be downloaded at: https://www.mdpi.com/article/10.3390/ijms24054274/s1.
