# Supplementary material for: Artificial Intelligence-Based Computational Screening and Functional Assays Identify Candidate Small Molecule Antagonists of PTPmu-Dependent Adhesion
Source: Int J Mol Sci. 2023 Feb 21;24(5):4274. doi: 10.3390/ijms24054274 (PMC10001486; doi:10.3390/ijms24054274)
Supplement: Supplementary file 1 [file ijms-24-04274-s001.zip › ijms-2083041-supplementary.pdf]

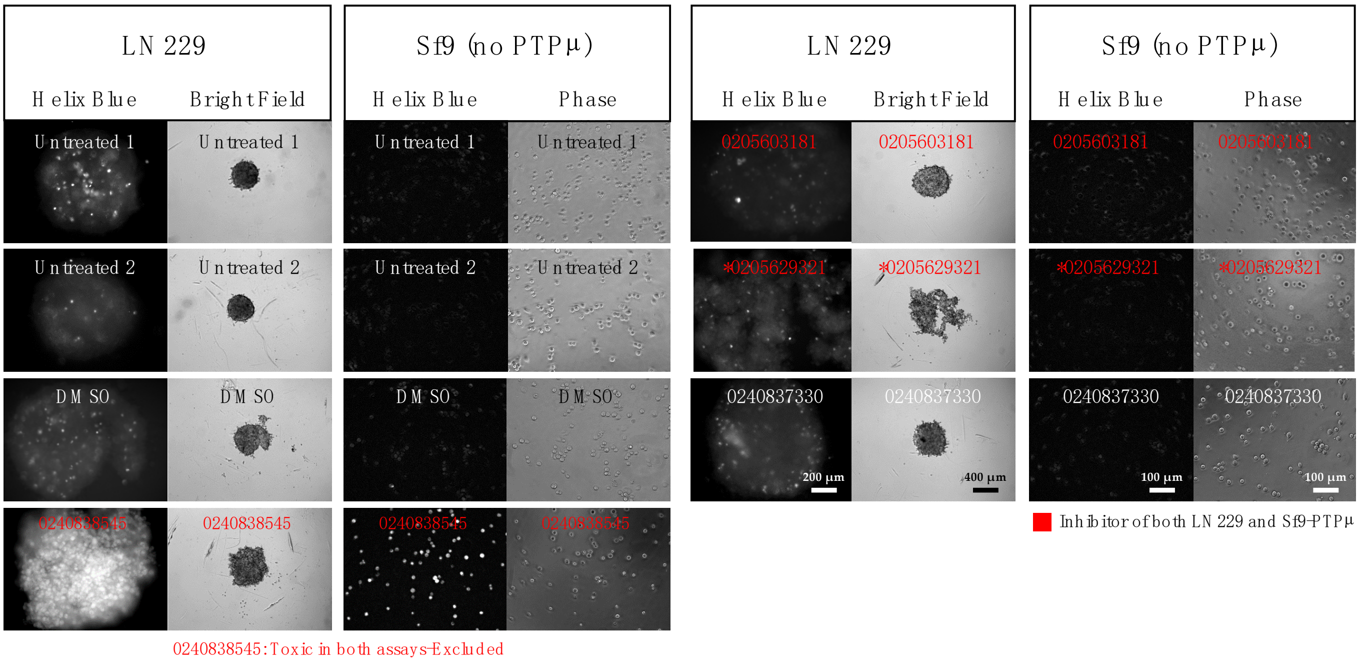

Figure S1. Examples of Helix Blue staining for cell toxicity. LN229 cells (plated onto non-adherent surfaces) or Sf9 cells were treated for 24 h with 100 μM of the indicated compounds. Two examples of untreated glioma spheres are shown to illustrate the varying degree of cell death present in normal spheres. The two priority compounds identified in our screen were not toxic to either cell type. One compound was found to be toxic to both cell types and was eliminated from the screen.

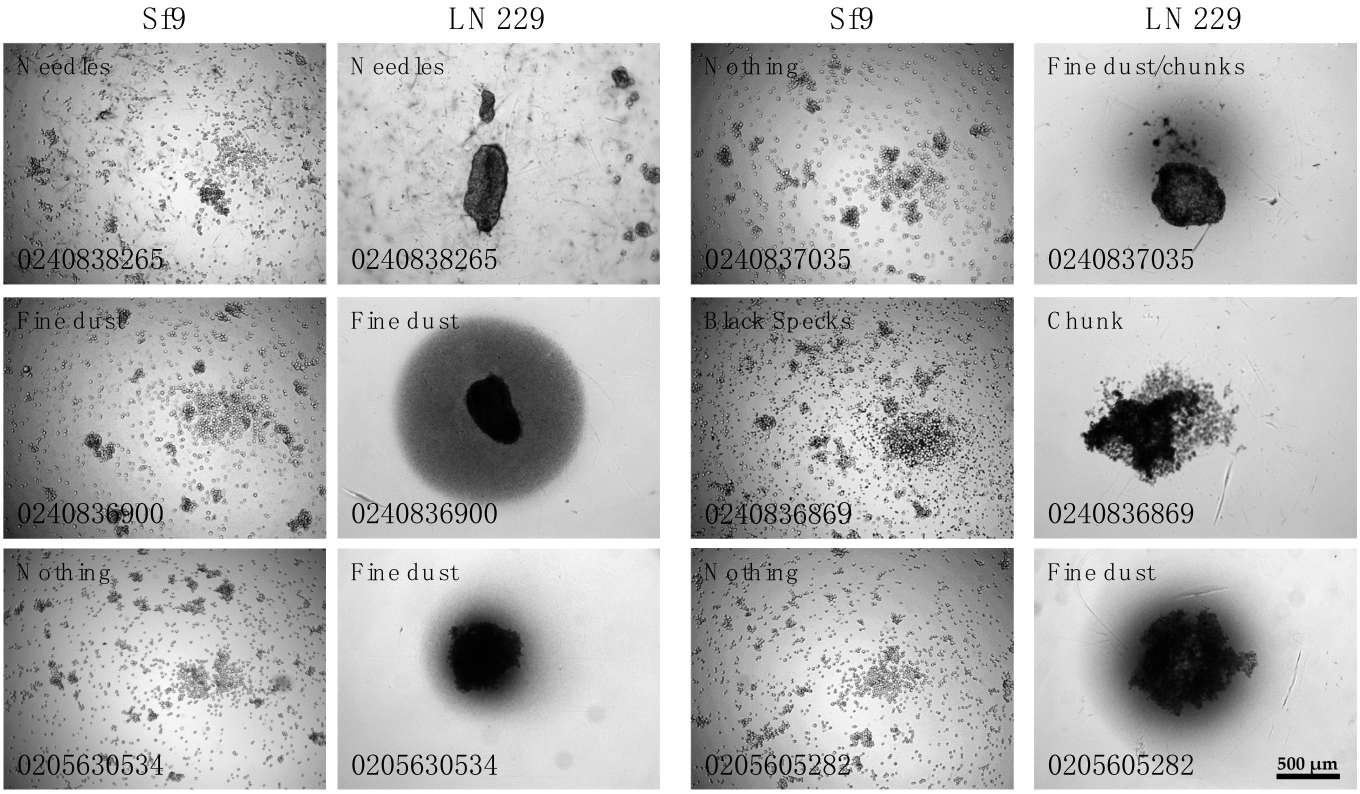

Figure S2. Examples of insoluble compounds. Eighteen compounds were eliminated due to signs of insolubility in either or both the aggregation assay and sphere assay. The appearance of precipitate varied depending on the compound.

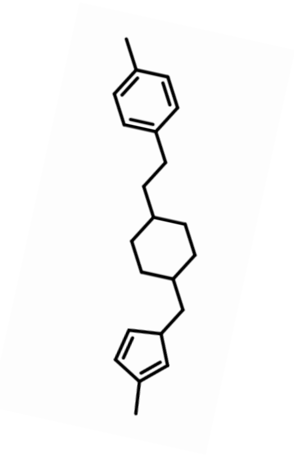

C S_0205629321

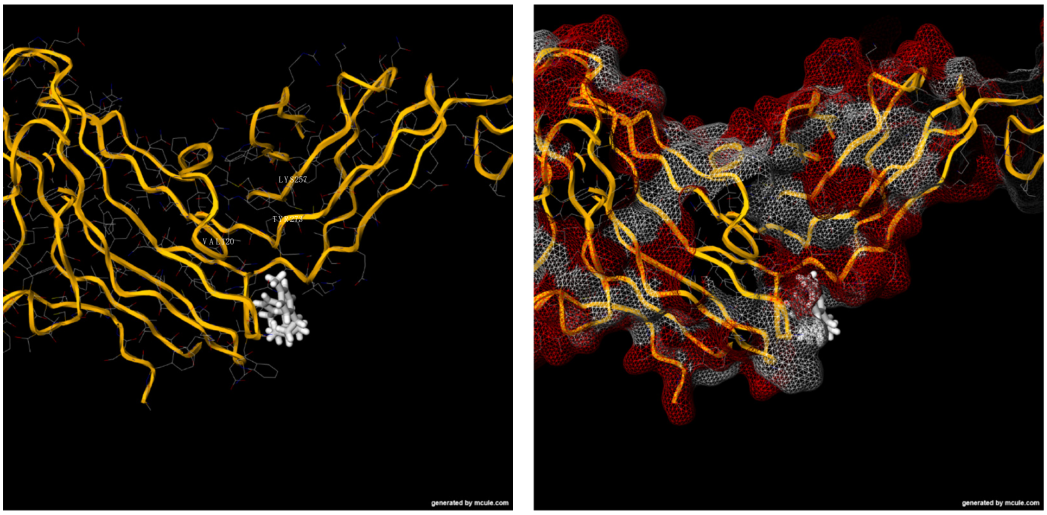

Figure S3. Structure and best predicted binding pose for a carbon substituted base structure (CS) of 0205629321. The CS of 0205629321 was docked with mCule 1-click docking (https://mcule.com/apps/1-click-docking/) using the crystal structure of the extracellular domain (Protein Data Bank IDs 2V5Y) and entry to the binding pocket defined as His187.

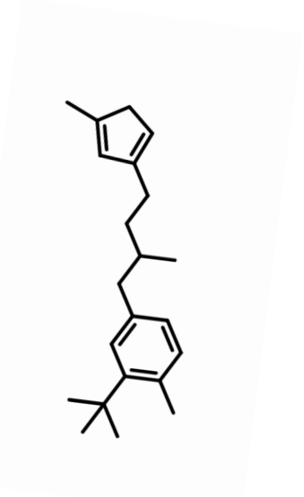

C S_0205603181

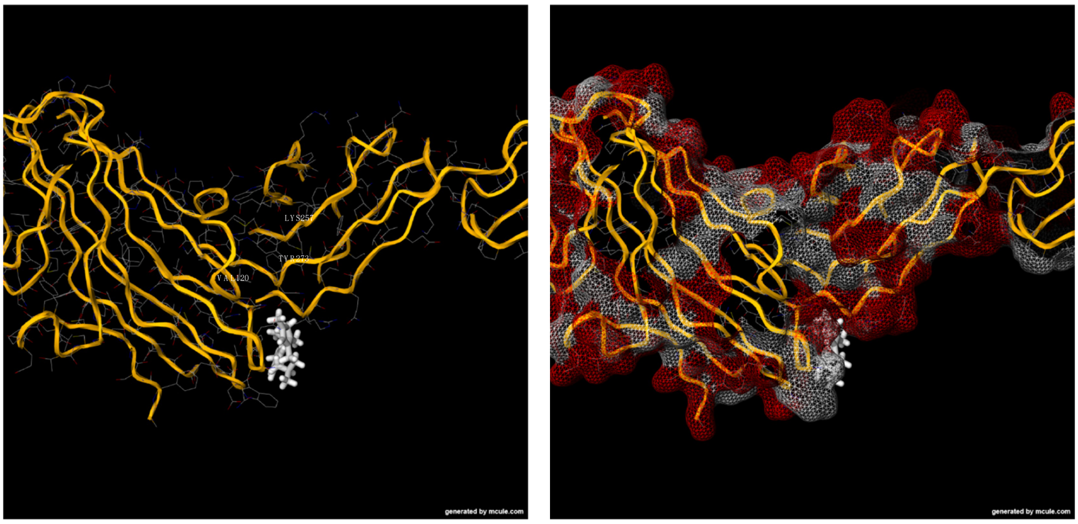

Figure S4. Structure and best predicted binding pose for a carbon substituted base structure (CS) of 0205603181. Atoms in the CS of 0205603181 were docked with mCule 1-click docking (https://mcule.com/apps/1-click-docking/) using the crystal structure of the extracellular domain (Protein Data Bank IDs 2V5Y) and entry to the binding pocket defined as His187.

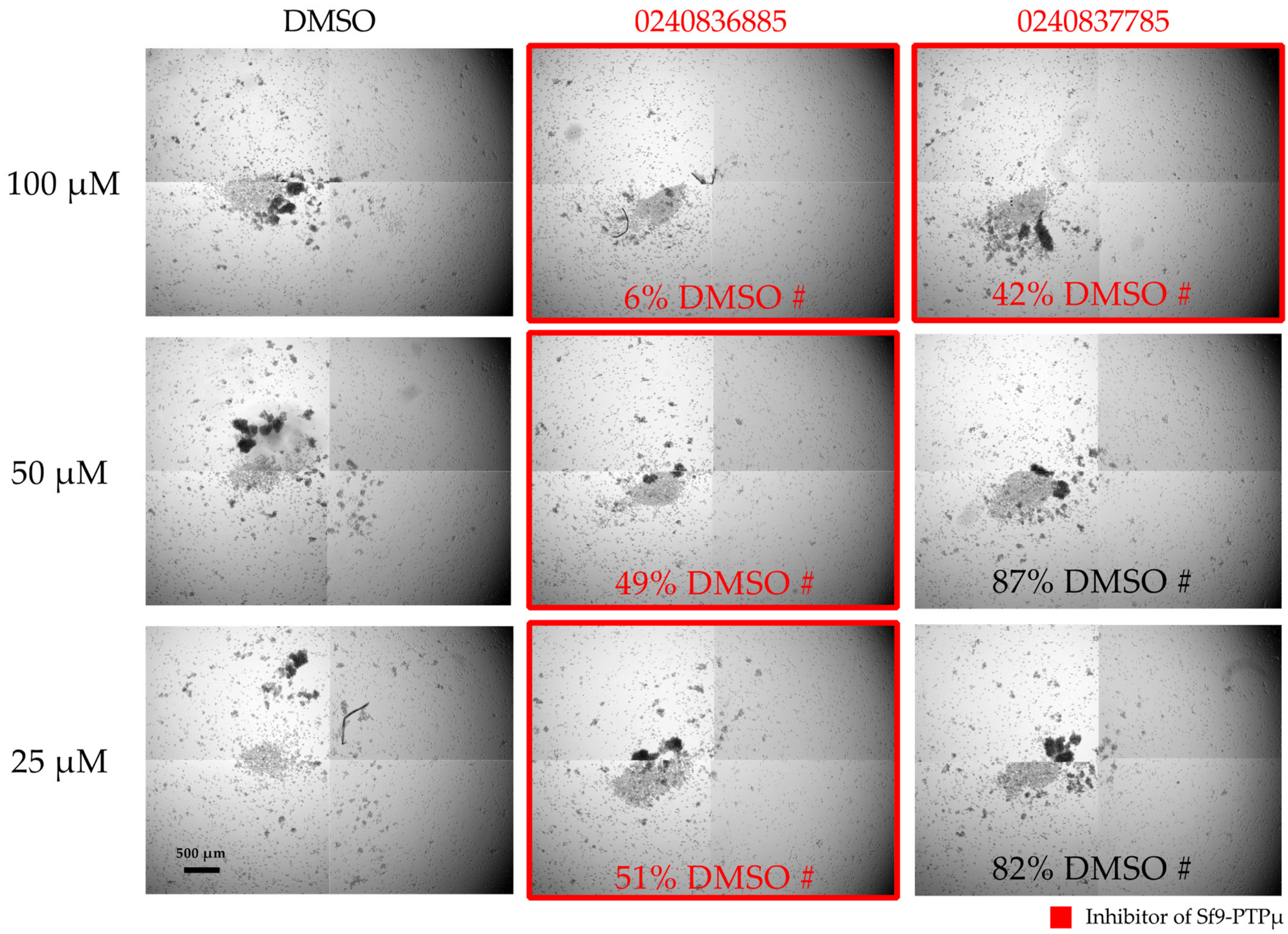

Figure S5. Titration of two compounds that inhibited PTPµ aggregation in Sf9 cells but did not affect glioma cell spheres. PTPµ-expressing Sf9 cells were treated with the indicated concentration of compounds for 20 min then rotated for 30 min to stimulate aggregation. Each well was imaged in its entirety as a 4x4 grid, but only representative central tiles are shown. The strongest inhibitor identified in the initial screen (Figure 2) was active down to 25 µM.

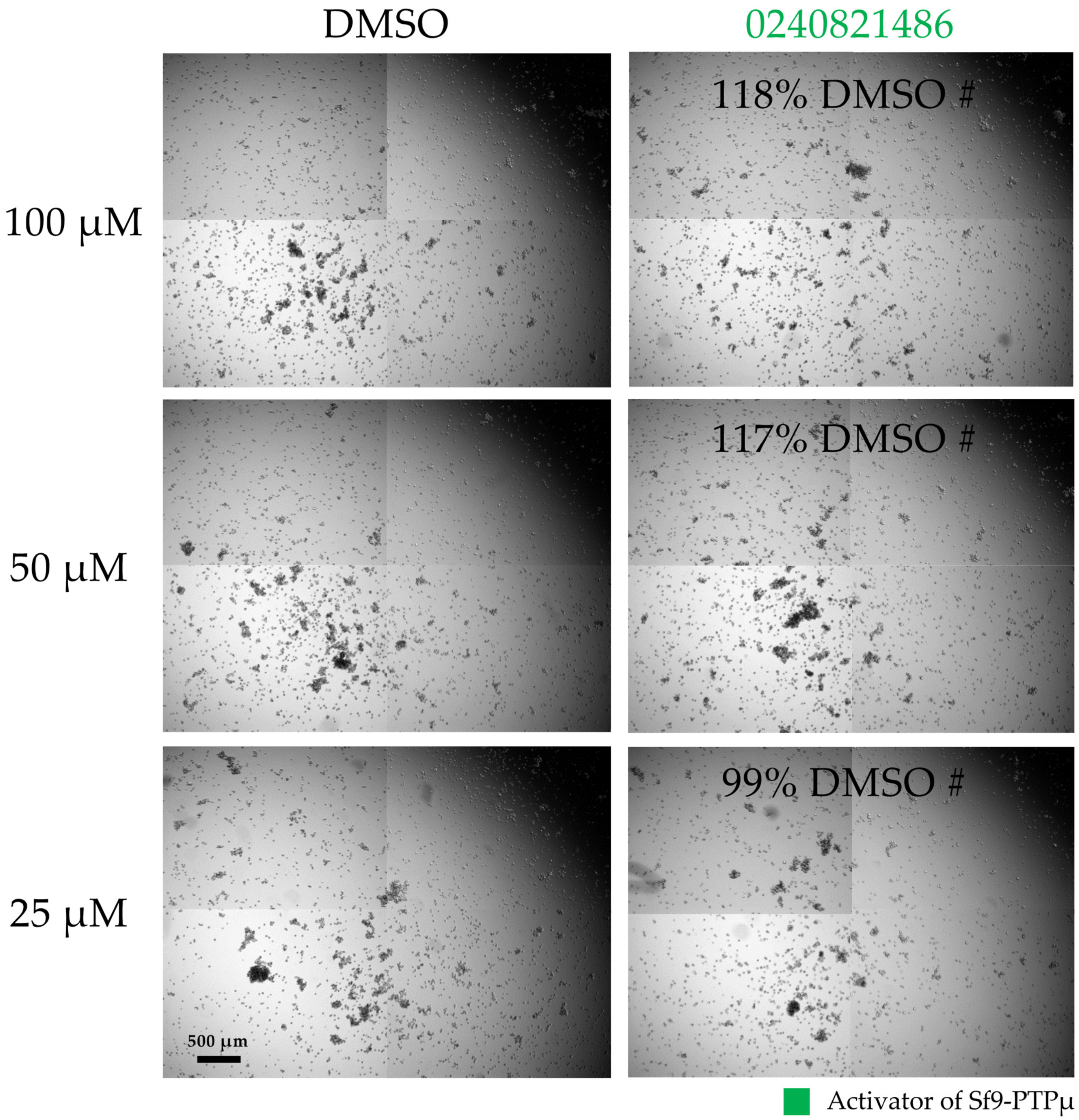

Figure S6. Titration of one compound that stimulated PTPµ aggregation in Sf9 cells but did not affect glioma cell spheres. PTPµ-expressing Sf9 cells were treated with the indicated concentration of compounds for 20 min then rotated for 30 min to stimulate aggregation. Each well was imaged in its entirety as a 4x4 grid, but only representative central tiles are shown. A potential activator identified in the initial screen (Figure 2) was weak on follow-up.

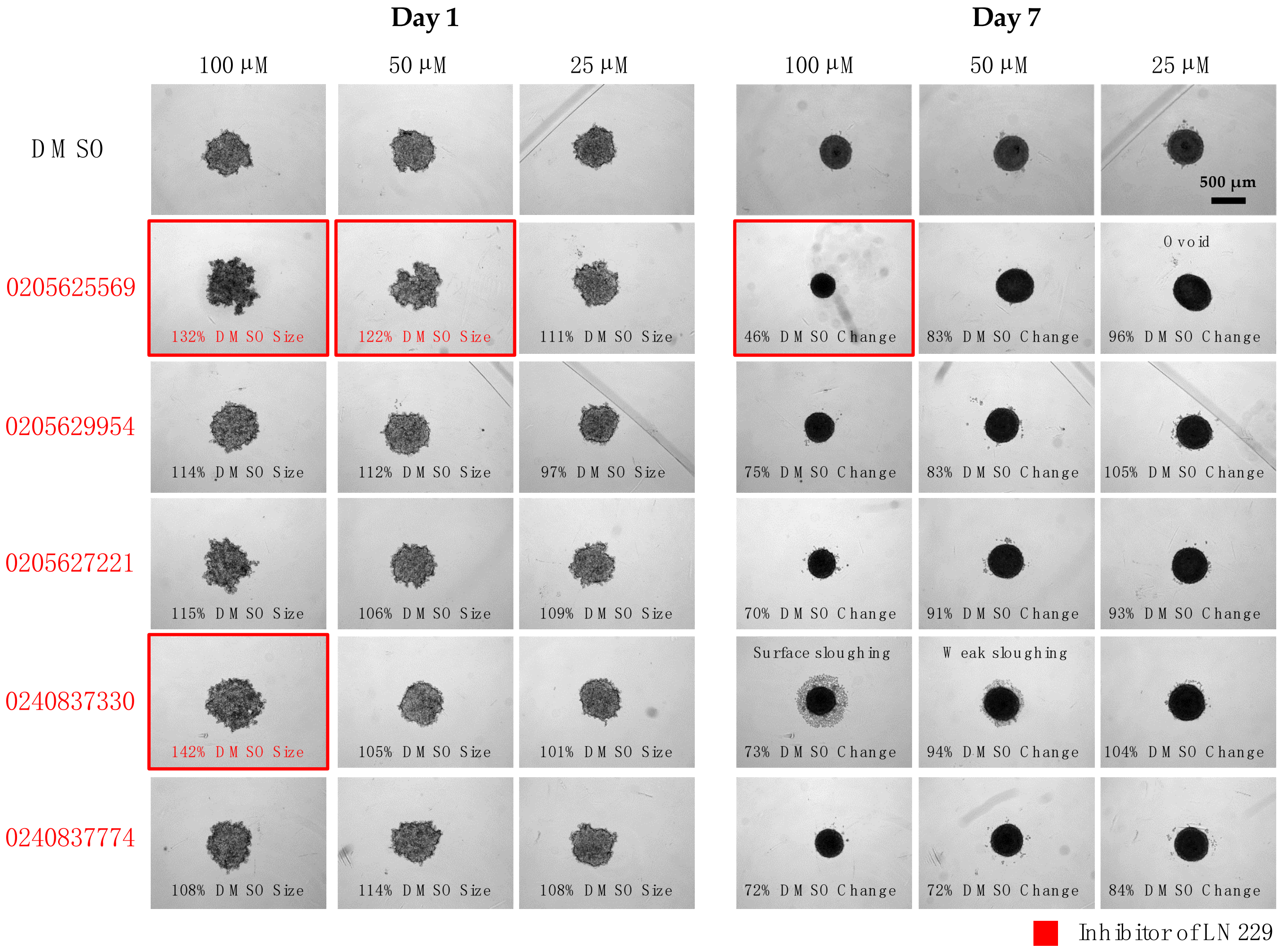

Figure S7. Titration of five compounds that inhibited glioma sphere formation/growth but did not affect PTPµ aggregation in Sf9 cells. LN229 cells were plated into non-adherent 96-well u-bottom plates and treated with the indicated doses of compounds. Sphere footprint sizes were measured on day 1 and sphere growth was measured on day 7. One compound inhibited sphere formation down to 50 µM and, at 25 µM, altered the shape of day 7 spheres. One compound inhibited sphere formation at 100 µM and caused surface sloughing of cells in day 7 spheres at 100 and 50 µM. The other three compounds had only modest inhibitory effects on sphere growth at 100 µM on follow-up.
